# Tuberculous Cervical Lymphadenitis Masquerding as Metastatis From Papillary Thyroid Carcinoma

**DOI:** 10.5812/ijem.4500

**Published:** 2012-06-30

**Authors:** Syed Mushtaq Saif Andrabi, Mohd Hayat Bhat, Bagdadi Farhana, Sameena Saba, Riyaz Saif Andrabi, Parvez Ahmad Shah

**Affiliations:** 1 Department of Medicine SMHS Hospital Srinagar, Kashmir, India; 2 Department of Medicine SKIMS Soura Srinagar, Kashmir, India

**Keywords:** Papillary Carcinoma, Lymphadenopathy, Tuberculosis

## Abstract

Clinically apparent cervical lymphadenopathy has been found at the initial presentation in 23 to 56 % of cases of papillary thyroid carcinoma. Here we report tuberculous lymphadenitis mimicking metastatic lymph nodes from papillary thyroid carcinoma and suggest that tuberculosis apart from metastasis in papillary thyroid carcinoma should also be considered in the etiology of enlarged lymph nodes in such patients, especially in those with risk factors for tuberculosis. Therefore, the importance of careful pre-operative evaluation of cervical lymph node metastasis cannot be overestimated, so that patients do not undergo unnecessary neck dissection for other benign conditions.

## 1. Introduction

Papillary thyroid carcinoma with cervical lymphadenopathy due to co-existent tuberculosis is common. Cervical lymphadenopathy is a common clinical manifestation in patients with papillary thyroid carcinoma and it has been shown that clinically apparent cervical lymphadenopathy can present as initial manifestation in about 23-56 % of cases (1, 2). As neck dissection can lead to numerous postoperative complications in these patients (3, 4), a proper pre-operative evaluation for the presence of neck metastasis is mandatory for these patients.

## 2. Case Report

**Figure 1:**
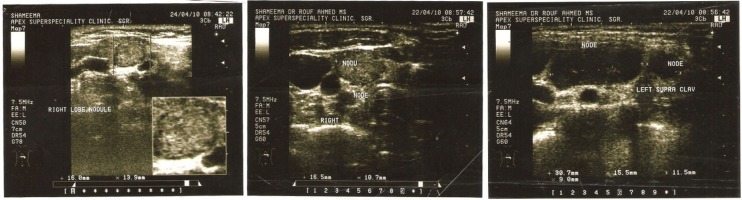
Ultrasonography of Neck Shows a Solid Thyroid Nodule. Ultrasound neck Showing a) Solid nodule in right lobe of thyroid b) Multiple enlarged right cervical lymph nodes c) Multiple enlarged lymph nodes in left supraclavicular region

**Figure 2:**
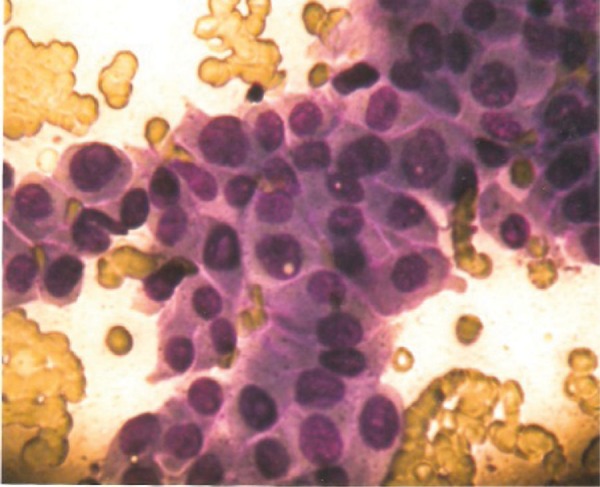
FNAC of Thyroid Nodule Showing Features of Papillary Thyroid Carcinoma.

**Figure 3:**
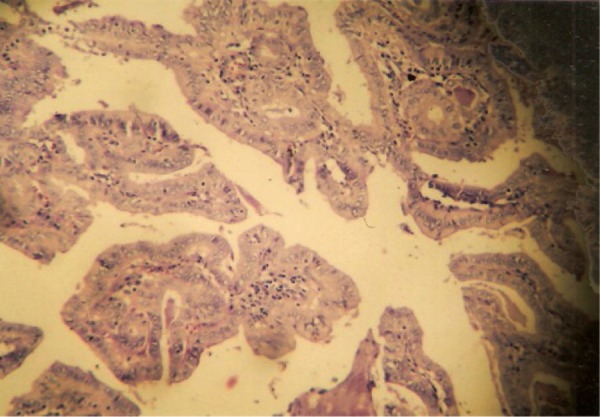
HPE Thyoid Nodule Confirming Papillary Thyroid Carcinoma

**Figure 4:**
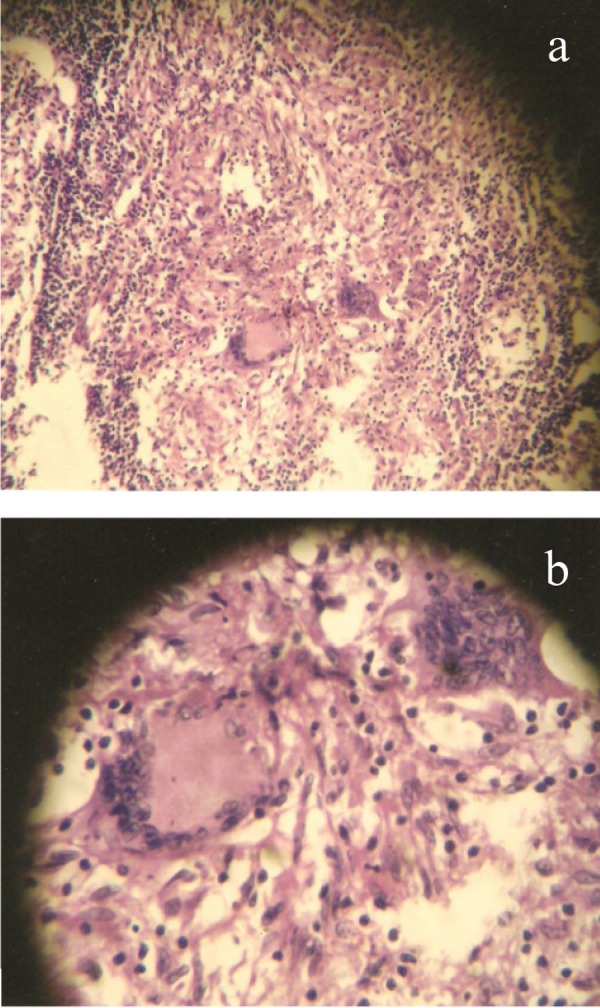
Section From Pretracheal, Supraclavicular and Cervical Nodes a) HPE of lymph nodes showing features of florid caseous tubercular lymphadenitis b) High power view showing epitheloid cells within lymph nodes

A 40 year old woman was admitted for evaluation of a thyroid nodule. She was a known case of type 2 diabetes mellitus from last three years on insulin therapy. She presented a history of decreased appetite and weight loss for last 2 months. General physical examination revealed a firm nodule in right lobe of thyroid without any associated bruit. There were also palpable lymph nodes in left supraclavicular area. She was clinically euthyroid. Investigations revealed normal Hemogram and serum biochemistry. Thyroid profile was also normal with TSH 4.7 mIU/L and FT_3_ 3.8 pg/ml (2.0-4.4). Ultrasonography of neck confirmed a solid thyroid nodule measuring 6.4× 6.8 mm in right lobe of thyroid with multiple bilateral cervical lymph nodes and nodes in the left supraclavicular areas of neck which was suggestive of metastatic neck nodes (Figure 1). Preoperative fine needle aspiration cytology (FNAC) was performed for the primary thyroid mass and neck nodes. Cytological examination of the thyroid nodule by fine needle aspiration cytology proved papillary thyroid carcinoma (Figure 2) and right cervical lymph node also revealed metastatic deposits in the form of dispersed cell with a secondary granulomatous reaction. Fine needle aspiration cytology (FNAC) of left supraclavicular node revealed epithelioid cell clusters with giant cells and reactive lymphoid cells suggestive of granulomatous lymphadenitis. Contrast enhanced computed tomography (CECT) chest showed Pretracheal and Paratracheal lymphadenopathy. The patient underwent total thyroidectomy and modified neck dissection with functional neck dissection on right side with suspicion of metastatic nodes. However, the lymph nodes were hard and severely adhered to adjacent internal jugular vein, which is not common metastatic characteristic. Histopathological examination confirmed papillary thyroid carcinoma of conventional and macro follicular morphology (Figure 3). No pericapsular invasion was seen. Section from all nodes (pretracheal, supraclavicular and cervical nodes) showed florid caseous tubercular lymphadenitis with no evidence of metastatic deposits (Figure 4). ^131^I whole body scan done after surgery revealed neck uptake with no metastasis elsewhere. Patient received 1 cycle of 88.5 mci^131^ I after surgery. We started patient on antituberculous medication consisting of isoniazid, rifampicin, pyrazinamide and etambutol after surgery. Patient is doing well on follow up (form last two and half years) with no recurrence of both the diseases.

## 3. Discussion

Papillary thyroid carcinoma with cervical lymphadenopathy due to co-existent tuberculosis is common. Cervical lymphadenopathy is a general clinical manifestation in patients with papillary thyroid carcinoma and it has been seen that clinically apparent cervical lymphadenopathy can present as initial manifestation in about 23-56 % of cases (1, 2). Recently Iqbal M et al. in his study showed that about 80 % patients of cervical lymphadenopathy with PTC were due to benign disease; out of which 72 % were due to tuberculosis and 8 % were due to reactive hyperplasia, and 20 % had metastasis in lymph node due to PTC (5). Neck dissection is generally indicated in papillary thyroid carcinoma patients with a clinically node-positive lateral neck. Although this procedure is reliable and relatively safe, considerable post-operative complications can occur (3, 4). Therefore, the importance of careful pre-operative evaluation of lateral cervical lymph node metastasis cannot be overestimated, so that patients do not undergo unnecessary neck dissection for other benign conditions. Ultrasonography is the most initial sensitive diagnostic method for detection of metastatic lymph nodes from Papillary thyroid carcinoma and the features which differentiate metastatic cervical lymph nodes from normal or reactive cervical lymph nodes are intranodal cystic necrosis, peripheral calcifications, absence of an echogenic hilum, minimum axial diameter of the lymph node > 7 mm for Level II and > 6 mm for the rest of the neck, hyperechogenicity in relation to the adjacent muscles, and the absence of an echogenic hilum (6). Among them, the most specific is the presence of cystic necrosis and calcification within the lymph node (6). Therefore, only in cases which shows such sonographic findings in cervical nodes of patients, metastasis from papillary thyroid carcinoma should be suspected before other benign diseases. In our patient no cystic necrosis or peripheral calcifications was found in the cervical lymph nodes on ultrasonography. Nevertheless, physicians should not overlook the discrimination of metastatic lymph nodes from tuberculosis lymphadenitis in patients with papillary thyroid carcinoma. The reasons are as follows:

1. Tuberculosis lymphadenitis is usually found in the supraclavicular area or the posterior triangle of the neck and is frequently the same site for metastasis from papillary thyroid carcinoma (7, 8).

2. Sonographic findings of tuberculosis lymphadenitis are very similar to those of metastatic lymph nodes in PTC patients. Sonographic features of tuberculosis nodes tend to be hypoechoic and round and usually show intranodal cystic necrosis and calcification similarly to metastatic PTC cervical nodes (9).

3. The treatment of choice for metastatic cervical nodes is neck dissection, which can lead to complications, whereas the treatment for tuberculosis lymphadenitis is anti-tuberculosis medication, which is far less complicated than surgery. Although necessary in endemic areas of tuberculosis, this differential diagnosis is also valuable in developed countries because of the increasing incidence of acquired immunodeficiency syndrome and associated tuberculosis (10).

In regard to this notion, although ultrasonography of the neck alone may be helpful in the differential diagnosis of abnormal cervical nodes, it is unlikely to be clinically useful to distinguish between metastatic lymph nodes resulting from papillary thyroid carcinoma and those caused by tuberculosis lymphadenitis. An additional method that can compensate for the low specificity of ultrasonography alone is FNAC (fine needle aspiration cytology) and its sensitivity has been reported between 46 % and 90 % (11-13). The preoperative FNAC of our patient revealed metastatic deposits in the form of dispersed cells with a secondary granulomatous reaction but histopathological examination revealed florid caseous tubercularlymphadenitis with no evidence of metastatic deposits (Figure 4).

Ultrasound-guided thyroglobulin in washout needle aspiration biopsy (FNAB-Tg) has been shown to be a valuable diagnostic tool adjunct to cytology for evaluation in patients with suspicious cervical lymph node (14).

In summary, although the distribution of abnormal cervical lymph nodes located around the supraclavicular or posterior triangle area of the neck and pre-operative sonographic findings of cervical lymph nodes in patients with PTC seem to have metastatic characteristics such as cystic necrosis and calcification, these clinical findings are never pathognomonic for diagnosis of metastatic PTC because of its similarity to tuberculosis lymphadenitis. Therefore, in case of suspicious for metastatic cervical nodes in pre-operative patients with PTC, combined FNAC with PCR for detecting mycobacterium tuberculosis should be employed for the differential diagnosis of tuberculosis lymphadenitis especially in endemic regions (15). In doing so; physicians may be able to limit the various complications associated with neck dissection. Tuberculosis is a common disease in developing countries and should be considered in the differential diagnosis of enlarged lymph nodes and properly evaluated even if metastasis seems to be an obvious cause for the same.
